# To What Extent Is General Intelligence Relevant to Causal Reasoning? A Developmental Study

**DOI:** 10.3389/fpsyg.2022.692552

**Published:** 2022-05-06

**Authors:** Selma Dündar-Coecke

**Affiliations:** ^1^Centre for Educational Neuroscience, Department of Psychology and Human Development, UCL Institute of Education, University College London, London, United Kingdom; ^2^Quantum Brain Art – QBA, Oxford, United Kingdom

**Keywords:** causal reasoning, explanation, development, domain-specific knowledge, general intelligence, fluid reasoning

## Abstract

To what extent general intelligence mechanisms are associated with causal thinking is unclear. There has been little work done experimentally to determine which developing cognitive capacities help to integrate causal knowledge into explicit systems. To investigate this neglected aspect of development, 138 children aged 5–11 studying at mainstream primary schools completed a battery of three intelligence tests: one investigating verbal ability (WASI vocabulary), another looking at verbal analogical (Verbal Analogies subset of the WRIT), and a third assessing non-verbal/fluid reasoning (WASI block design). Children were also interviewed over the course of three causal tasks (sinking, absorption, and solution), with the results showing that the developmental paths exhibited uneven profiles across the three causal phenomena. Children consistently found that explaining solution, where substances disappeared toward the end of the process, was more challenging. The confirmatory factor analyses suggested that the impact of cognitive ability factor in explicitly identifying causal relations was large. The proportion of the direct effect of general intelligence was 66% and it subsumed the variances of both verbal measures. Of this, 37% was the indirect effect of age. Fluid reasoning explained a further 28% of the variance, playing a unique role in causal explanation. The results suggested that, overall, cognitive abilities are substantially related to causal reasoning, but not entirely due to developmental differences in “*g*” during the age periods studied.

## Introduction

Any credible form of human thinking corresponds one way or another to causal reasoning. We have a tendency to explore what causes an effect, and whether the same effect consistently follows the same cause (Woodward, 2003)—when it does, we can therefore establish a link between the two events that can be legitimately explained.

Causal explanations typically offer a mechanism that connects an effect to its cause in a lawlike relation. For instance, in order to hold the view that “Gravity causes objects to rest on the ground,” we would potentially rule out all other non-causal influences and fit the best explanandum into a causal nexus that is hard to vary in different situations. Several forms of cognitive activity are involved in the identification of such relations, whether in academic settings, such as when investigating a phenomenon in a school science lesson, or at a fundamental level when simply trying to understand events in everyday life.

Despite its importance, how our capacity for such survival reasoning evolves is largely unknown. In particular, which developing cognitive abilities help to represent and build causal knowledge into explicit systems is unclear. This leaves us with a major question in mind: do developmental paths vary solely due to individuals’ general cognitive abilities, or are there other factors at play in explicitly defining causal relations? For instance, although evidence is robust that by their third birthday the majority of children can formulate some sort of causal explanation as to why a cause produces the observed effect (e.g., Callanan and Oakes, 1992); and that explanatory capacities develop over the course of childhood (Carey, 1985; Legare and Clegg, 2014; Lombrozo and Vasilyeva, 2017; Busch et al., 2018), which aspects of intelligence develop and contribute to such thinking is largely unknown.

The aim of the present study is to explore the sources of variation in causal explanatory thinking. We ask how strong the relationships between cognitive ability systems and causal explanatory performances are across the development. For such investigation, two epistemological routes examining the nature of *change* in reasoning and better handling are relevant: the psychometric (/differential) and the developmental route (see Demetriou et al., 2010). The psychometric theories analyze the aspects of variation in mental abilities and expound the ways in which the processes underlying individual differences are reliably understood and measured (see Spearman, 1927; Eysenck, 1967; Sternberg, 1977a,^1978^; Kyllonen and Christal, 1990). In contrast, developmental theories take an expansive approach to the study of the notion of *change* in cognitive, behavioral, and societal contexts. Unlike the psychometric route, the impacts of variation in causal thinking have been investigated broadly by developmental work as discussed below.

### The Psychometric Route

Psychometric theories concede that intelligence is an open-ended category for several cognitive mental operations and steers most core competencies, including perception, attention, learning, and memory. Although there is no widespread consensus as to what intelligence is, most cognitive tests tend to cluster in a number of broad ability factors, such as *fluid* (*gf*) and *crystallized intelligence* (*gc*) and hence, studies from diverse strands commonly employ well-known standardized psychometric measures for evaluation. The crystallized form of intelligence is commonly captured by verbal comprehension and vocabulary measures, whilst the fluid form is usually measured *via* non-verbal and procedural reasoning tasks. The interrelation between these broad abilities also points to the existence of a higher order common factor that Spearman (1904) named *g*. The present study takes into account this hierarchical structure, as discussed below, where g stands as a latent variable, existing as a summative index linking these ability factors.

When Spearman (1904) realized that most cognitive tests correlate with each other, he devised a technique, called factor analysis to analyze the sources of variation common to performance in all cognitive tests. This analysis revealed two types of factor, namely the general (g) and specific factors (s). He used the term “positive manifold” to refer to the existence of *g* as representing the portion of variance that existing intelligence tests have in common, proposing that people who score well on one cognitive task are likely to do well in others. Since then, the psychometric *g* has been used as an index or a score factor derived from cognitive tests, standing for a latent variable—a sum total of an individual’s scores (Scarr, 1997; Jensen, 1998, 2002).

Cattell (1943, 1971) and Horn (Cattell and Horn, 1978) analyzed the nature of *g* by breaking it down into two major cognitive constructs, namely fluid (*gf*) and crystallized intelligence (*gc*). This approach, for the first-time, theorized *g* as composed of more than one factor, where the broad second-order factors like *gf* and *gc* constitute the top stratum and are based on more than 40 first-order factors that forming the lower stratum. The first order factors accounted for by specific abilities (*s*) refer to diverse and specific cognitive competences (Cattell, 1971; Hunt, 1997; McArdle et al., 2000).

Carroll’s (1993; 1996) three-stratum theory of intelligence presented these cognitive factors in hierarchical terms and theorized a multifactorial taxonomy responsible for the variability in cognitive performance. In this hierarchy, the top stratum, conceptually equivalent to Spearman’s *g*, strongly correlates with eight factors placed at the second level that are differentially influenced by *g*. These broad abilities, similar to Cattell and Horn’s theory, include fluid intelligence (*gf*), crystallized intelligence (*gc*), analogical reasoning, and working memory, which are then broken down into a number of specific abilities within the third stratum (see also Horn and Cattell, 1966; Jensen, 1998; Deary, 2001; Carroll, 2003; Sternberg, 2003).

In addition to these hierarchical models, several studies have contributed to the diverse analysis of intelligence, with recent theories dealing with, for instance, biological aspects of the brain and neural functioning (Reed and Jensen, 1992); cognitive correlates between laboratory-based cognitive tests and psychometric intelligence tests (Cronbach, 1957; Hunt et al., 1973; Sternberg, 1977b); heritability (Loehlin, 1989); and the bioecological basis of individual differences, taking into account the environment and context in cognitive performance (Ceci, 1996).

An integrative model bridging between the psychometric and developmental routes proposes four types of mental processes: (1) domain specific (verbal, numerical, etc.), (2) representation processes (memory, updating, etc.), (3) reasoning (inductive, causal, etc.), and (4) cognizance (common sense, morality, etc.). Although all these processes are intertwined with g, what varies is the strength of the relations between them and their contributions to the executive processes over major developmental cycles (see Demetriou et al., 2017). Such psychometric assessments provide consistent outcomes for underlying individual differences responsible for cognition, adaptation to environment, and novel problem solving (Sternberg, 2003). However, despite the broad range of cognitive abilities studied in this literature, causal reasoning has never been a distinct focus, leaving us with a gap in knowledge around the psychometric aspects pertinent to such reasoning, particularly the relevance of *g* factor. Thus, here we ask; to what extent is *g* relevant to children’s causal explanations?

### The Developmental Route

Studies examining the link between cognitive development and causal explanatory thinking are diverse, but can be studied under three strands. The first strand was pioneered by Piaget (1930, 1936, 1959, 1974), who studied children’s causal reasoning about real-world phenomena across a wide range of knowledge areas, and proposed that causal reasoning is operated by domain-general knowledge. Such knowledge is accumulated gradually through progressive equilibration of divergent and initially specific constructs, during which a cognitive shift in children’s mental operations occurs, proceeding through an invariant sequence of *stages*. In each stage, children integrate an understanding of the dimensions of causal phenomena. For instance, if the quality of explanation of phenomena differs during early years, this occurs largely as a consequence of the child’s lack of operational thinking and accompanying language skills; and so it follows that, during the preoperational stages, children’s explanations are largely pre-causal, until genuine causal understanding appears around the age of 7/8 (Piaget and Inhelder, 1974).

In line with this Piagetian stage-wise progression approach, causal graphical models successfully instantiated the ways in which children develop a succession of different conceptions of the world, evolving from concrete individual experiences into abstract knowledge structures, growing increasingly accurate with time (Spirtes et al., 1993; Gopnik and Wellman, 2012). Furthermore, probabilistic and Bayesian frameworks made computational learning ideas available to developmentalists, with structured models that employ a top-down route in which causal schema is inferred from observable patterns (e.g., Gopnik et al., 2004; Griffiths et al., 2010, 2011). Cheng’s (1997) study, for instance, showed that people can infer causal relations from even a single event with a high level of abstraction. Developmental studies correspond with these arguments, as shown by Schulz and Gopnik’s (2004) study, demonstrating that children cross the domain boundaries when they need to distinguish between screening-off trials using patterns of independent and dependent probabilities across a range of domains, suggesting that formal causal reasoning mechanisms are general in nature (see also Gopnik et al., 2001; Tenenbaum and Griffiths, 2001; Sobel et al., 2004).

Despite the wealth of evidence, a significant question remained unresolved in this literature: how strongly are causal explanations linked to general cognitive abilities across development? In response to this question, the domain-specific approach gained momentum, with advocators of this strand arguing that causal knowledge grows *via* specific learning mechanisms which operate based on a range of unique inputs and structural principles, pointing to distinct ways of acquiring knowledge (e.g., Baillargeon, 2002). Just as the stomach is a specialized mechanism for the chemical breakdown of food—distinct from other alimentary canals such as the mouth and intestine-, specific cognitive abilities aid the handling of different kinds of information, such as language processing or visual systems, each of which, to some extent, develops semi-independently or independently (Keil, 1989; Hirschfeld and Gelman, 1994; Sobel, 2004).

At a preschool level, young children’s inadequacy with causal explanatory systems was explained by a lack of domain-specific knowledge, rather than the maturity of general ability alone. For instance, a child’s conception of “animal” changes when the child develops a theory-like biological domain about animals (Carey, 1985; Inagaki and Hatano, 2002). Although children’s theory-like knowledge systems are immature, these systems seem to provide them with the required causal devices enabling them to predict and explain phenomena in their surroundings. From preschool onward children can utilize these devices more efficiently, as they develop readily available causal explanations for various phenomena (e.g., contamination-based), even when unseen mechanisms (e.g., germs) need to be considered (Wellman and Gelman, 1998; Keil et al., 1999; Legare et al., 2009).

Further evidence supported the view that domain-specific knowledge facilitates understanding in folk psychology (Wellman, 1990), folk physics (Bullock et al., 1982; Vosniadou, 2001), and folk biology (Hatano and Inagaki, 1994; Gelman and Opfer, 2002; Inagaki and Hatano, 2002), following a bottom-up route where causal relations are layered and filtered *via* their common elements. Furthermore, infant studies found evidence that young children tend to consider domain specific mechanisms when they assess causal regularities. For instance, psychological phenomena occur *via* different causal principles (e.g., action at a distance), as opposed to most physical phenomena, where causal relations are observed from contact or *via* transference (Spelke et al., 1992; Carey and Spelke, 1996; Gottfried and Gelman, 2005; see also Leslie and Keeble, 1987). Although these studies argue that even infants can make inferences about causal events by taking into account basic physical principles, such as cohesion, continuity, or contact (Spelke, 1994), we need to bear in mind that these infant studies largely focused on perceptual processes rather than explanatory.

The third strand of thought offers an analysis beyond domain generality/specificity, with slightly different ideas, scrutinizing how children build causal theories about the world (see Gelman and Kremer, 1991; Karmiloff-Smith, 2015). In this line of thought, the role of executive function gains momentum, emphasizing the way in which knowledge is restructured during development *via* inhibitory control. Such analysis proposes that children are natural and active causal explanation-seekers; they continuously change conceptual structures of their naïve theories and renew their understanding of phenomena over the course of development by inhibiting previous misconceptions (Carey, 1985; Legare, 2014; Vosniadou, 2014; Zaitchik et al., 2016).

In line with this argument, recent accounts have acknowledged the role of executive function—particularly processing capacity—viewing it as a robust way of explaining the cognitive growth mediating the stage transitions (see e.g., Case, 1991; Demetriou et al., 2010). It is argued that mental processing mechanisms such as processing speed, executive control, and working memory, guide cognitive development, and mature around the 6–10 years period, where rule-based thoughts emerge and are established (e.g., Kuhn, 2000; Spanoudis et al., 2015; Demetriou et al., 2017). In contrast, descriptive ability emerges much earlier, as children can describe causal sequences without acknowledging the rules and laws involved. However, the development of causal explanatory competences is boosted by the emergence of rule-based thoughts, where underlying causal connections are drawn from seen and unseen aspects of mechanisms (Demetriou et al., 2010; Kuhn, 2010).

The literature therefore presents several methods of evaluation *via* which the development of causal explanatory thinking can be analyzed. One way of advancing our understanding about the possible contributors to children’s causal explanations is to conjecture the developmental trajectories across a number of phenomena comparatively with general intelligence measures. The present study aims to explore this avenue as discussed below.

### The Present Research

In a broad context, we aim to understand which developing cognitive factors are affecting the quality of children’s explanations when they talk about causal phenomena, and how such cognitive abilities might influence each other as they advance. Two research questions aim to investigate this target as below.

(1) Does the kind of phenomena present a greater or lesser challenge to the children?
(2) If any, which general intelligence mechanisms are associated with the variance in causal reasoning, specific to the age period examined?

It is hypothesized that children can construct similar levels of explanation when faced with different causal tasks. If this is not the case, to what extent the source of variability in children’s causal explanations is associated with their psychometric performance across different causal phenomena is the concern.

Children’s causal judgments were obtained *via* three causal tasks—sinking, absorption, solution, analogously representing physical, biological, and chemical domains. Previous studies showed that children can acquire causal knowledge from the observation of such natural phenomena typically studied in elementary school science and encountered in everyday life. Such causal tasks require children to engage with mechanism-based thinking, in which underlying causal mechanisms need to be inferred from observable and unobservable structures (Dündar-Coecke et al., 2019, 2020, 2021).

The level of children’s explanation was evaluated using a robust stratified model, with causal task interview protocols kept identical in each task as explained in the methodology section. The procedure followed the general pattern of the predict-observe-explain (POE) methodology—a pedagogical approach that is widely used in science teaching (see White and Gunstone, 1992), where participants first predict an outcome, then observe and explicitly describe what they witness, and then explain why things occurred the way they were observed.

The purpose of the prediction stage is to capture children’s counterintuitive ideas about phenomena. In the description stage, the aim is to capture children’s ability to present a contextual description of observed phenomena. For instance, in the context of sinking, children are expected to produce descriptive remarks about sinking rather than flying. Finally, the purpose of the explanation stage is to capture children’s interpretations of factors and variables involved in causal mechanisms. For example, when explaining sinking, how children infer factors, such as weight, operating as variables in the determination of sinking rate (e.g., weight as downward direction; up-thrust as upward direction) is the concern.

Cognitive ability measures detected different psychometric properties as verbal, analogical-verbal (i.e., *gc*), and non-verbal abilities (i.e., *gf*). Associations between each of these constructs were analyzed using parametric and confirmatory factor analyses as explained below.

## Methodology

### Design

The present study combined the cross-sectional and individual differences approaches and included 3 year groups spanning the English elementary school age range, namely Year 1, 3, and 5.

### Participants

The initial sample included 140 typically developing children who volunteered to take part, from two primary schools located in Oxford, United Kingdom. For the recruitment process, parents signed a consent letter to allow their children’s participation. Children’s verbal consent was also obtained before each session following the UCL Institute of Education Research Ethics Committee’s ethical requirements. Two children’s data were excluded from the analyses due to lack of interest after the start of testing. Therefore, analyses included 138 children covering a wide range of socioeconomic backgrounds [67 girls and 71 boys; 47 Year 1 (Y1), mean age = 6.1, *sd* = 3.7 months; 45 Year 3 (Y3), mean age = 8.3, *sd* = 4.6 months; and 46 Year 5 (Y5), mean age = 10.01, *sd* = 6.2 months]. As the parents’ questionnaire showed, 37.6% children came from bilingual/trilingual homes, which can be taken as an indicator that the sample included a moderate ethnic and linguistic variety.

### Measures

#### Standardized Cognitive Measures

The expressive *vocabulary* subtest from the Wechsler Abbreviated Scale of Intelligence (WASI-vocabulary) (Wechsler, 2011) was used to provide measures of verbal (crystalized) intelligence. WASI vocabulary is a measure of expressive language, word knowledge, and verbal concept formation. Each word was read aloud by the experimenter and children were asked to describe and explain the words with their meanings. Administration and scoring followed standard procedures.

The WASI *Block Design* is a subtest to explore children’s non-verbal intelligence (Wechsler, 2011). Children were shown nine red and white square blocks and a spiral booklet of cards showing different patterns, and the children were asked to imitate the patterns using the blocks, with the exercises increasing in difficulty at each stage. The Block Design aimed to measure children’s fluid reasoning and ability to analyze/synthesize abstract stimuli within a specified time limit. Again, administration and scoring followed standard procedures.

The *Verbal Analogies* is a subset of the Wide Range Intelligence test (WRIT). It is a brief measure of verbal intelligence designed to evaluate children’s and adults’ verbal reasoning, and receptive language ability, as well as short-term memory, between the age range spanning from 4 to 85 (Glutting et al., 2000). This test requires participants to provide an appropriate word/concept to complete an analogy sentence (e.g., “The sky is to blue as snow is to …”). Administration and scoring followed standard procedures.

#### Causal Tasks

Each task involved the demonstration of two contrasting examples to make causal processes more salient to observers. Confirmatory, past research found that contrast demonstrations could significantly improve children’s conception of causal mechanisms (e.g., Namy and Gentner, 2002; Dündar-Coecke et al., Submitted).

For s*inking*, children saw a stone and a grape of similar size and color but of different densities, which sank at different rates in a 50-cm-high transparent jar of water. Initially, children inspected the contrast materials by touching and lifting, and (1) were asked to *predict* what they thought would happen if the items were dropped into the water together (prediction). They then (2) watched the focal events and were asked to *describe* what they had noticed during the demonstration (description), and (3) *explain* why they thought things had happened in the way that they had seen (see Supplementary Appendix 1 causal task scripts).

For *absorption*, children saw water rising from a petri dish through two strips of paper: a piece of tissue paper, through which the water rises faster due to the empty spaces between its cellulose fibers; and the same length and width of blotting paper, which has smaller empty spaces thus relatively reducing its absorbency. This task had the same three-stage structure as sinking, in which children: (1) predicted; (2) described; and (3) explained the causal relations (see Supplementary Appendix 1 causal task scripts).

For *solution*, children saw the same small quantities of table and rock salt dissolve in warm water. The greater surface area to volume of the table salt led to more rapid solution. Similar to the sinking and absorption protocols, children needed to (1) predict outcomes before witnessing simultaneous demonstrations of the two instances. They were asked (2) to describe the process they witnessed, and (3) explain the outcomes (see Supplementary Appendix 1 causal task scripts).

### Procedure and Analysis

Testing started with the administration of the WASI expressive vocabulary measure, followed by the Verbal Analogies (WRIT), and the WASI non-verbal Block Design tests. Administration of the cognitive tasks was followed by the three causal tasks, in the order of sinking, absorption, and solution. The initial start with cognitive ability measures aimed to warm up and encourage children to be more expressive in their explanations. Children were told that they could take a break or withdraw any time they wanted.

Each child saw two contrasting instances for each phenomenon, following the same administration protocol within a single one-to-one session. Children were not given any clues about the variables. There were no practice trials, and they did not receive feedback during the sessions, in order to minimize the learning effect and maximize reasoning competences with or without reliance on prior knowledge. Testing took place in a private room, or out of class in a quiet area within the schools. Each child took an average of approximately 33 min to test and complete the battery (min = 25; max = 47 min). All responses were recorded manually on the relevant score sheets for later analyses.

The data were analyzed using R and EQS structural equation modeling software. For the parametric tests, the reliability of the composite causal measures was tested using *Cronbach*’*s* α. The developmental trajectories were initially examined using ANOVAs. Since there was a significant skew on scores for non-verbal block design measure, the Welch robust index was used to test statistics for the cognitive measures. Main effects and differences between each age group were also assessed using Bonferroni corrections. The correlations were assessed using Pearson and partial correlations. To test the presence of possible multivariate outliers, Mahalanobis distance statistics were calculated for each variable. No data surpassed the *p* > 0.01 criterion, indicating that none of the variable values influenced relations among other variables. Estimates of the unique variance in each causal measure accounted for by each of the predictors were examined using hierarchical linear regression, with adjusted *R*^2^ values reported for the variance explained in each model. All tests were two-sided, with *p* < 0.05 observed power for ANOVA was 0.99, for regression analyses was 0.98, which were calculated using G-Power 3.1.9.2 (Erdfelder et al., 2007).

The EQS structural equation modeling software was used to test the relational magnitudes between cognitive and causal measures. In search of the best model, six models were examined. In the best model shown in Figure 2, a latent factor was built for each of the three causal reasoning measures standing for causal reasoning. The relation between this measure and each corresponding factor was fixed to 1; the error variance was left free to be estimated. Similarly, each dummy cognitive measure was regressed on a latent variable—a common factor standing for *g*, while the three causal measures were regressed on causal reasoning factor. Age was regressed on *g* and causal factors, and the causal factor was regressed on the residual of *g* rather than on *g* itself. Regressing causal factor on the residuals of the factors and *g*, rather than the factors themselves, allowed us to capture how, if at all, each reasoning process is uniquely related with causal reasoning in addition to their common components captured by *g*.

This model was based on Tucker-Drob’s (2009) standardized solution model, which is constructed based on an integration of Cattell-Horn’s approach, where *g* and broad abilities, such as *gf* and *gc*, were presented in a hierarchical structure. The Tucker-Drob model investigates the relations between the second stratum abilities and the higher order common (g) factor which associates largely with various abilities.

To test the goodness-of-fit, the following indices were used: (1) the χ*^2^* goodness of fit: the fitness of the model improved as the χ*^2^* value lowered. (2) Bentler’s *AIC* value: the model with the smallest *AIC* value provided the best fit. (3) The comparative fit index (*CFI*): compared the fitness of the model with the hypothetical one in which none of the variables are correlated. A model with the *CFI* value of 0.90 or higher means that the tested model fits the data well (Bentler, 2006). (4) The root mean square error of approximation (*RMSEA*) was used to test whether the data fitted the hypothesized model, with smaller values indicating better model fit. The recommended cutoff value is suggested usually to be smaller than 0.06 (Hu and Bentler, 1999).

### Scoring of Causal Tasks

Two kinds of scoring system were computed as below.

*1. Individual scores for prediction, description, explanation*: predictions (0–2) and descriptions (0–2) were scored for accuracy in reporting different sinking/absorption/solution rates for each phenomenon. The measures of prior knowledge and description made it possible to assess to what extent the level of explanation was a function of prior knowledge vs. a result of current observation. Therefore, the score given to prediction is in relation to whether children can present a reasonable estimation from prior knowledge. The score given to description is to capture the relevance of the description, i.e., how well it fits with the observation.

Explanations were scored using a stratified model, with responses given incremental scores between 0 and 3 as exemplified in the Supplementary Appendix (see causal task scoring system and examples for the level of explanations). This began with 0 for no explanation. Children who identified only one relevant factor (e.g., weight for sinking, softness for absorption, and size for solution) without comparisons received a score of 1 (e.g., “They are heavy, they sank to the bottom”). Children who identified the plural items and compared their variation received a score of 2 (e.g., “The stone was heavier than the grape, it sank to the bottom faster”). Children who identified any intervening factor relevant to the causal mechanism beyond the comparison score of 2 received a score of 3 (e.g., “Both stone and grape are heavy that water cannot push them up.” “But the stone is denser than the grape, it sinks faster”).

In this stratified system, scoring was blind to the children’s age. The criteria for judging a good explanation were not the number of sentences or the length of the sentences; rather, the methodology was guided by the level of the explanation. For instance, after observing sinking patterns of a stone and a grape, if a 5-year-old says, “The stone is heavier, has more stuff in it that makes it more compact, but the grape is not,” this was counted as corresponding to the highest-level explanation without use of the word density. Qualitatively, this explanation differs from the one concluding, for instance, “stone is heavier than the grape.” The former reflects the understanding and coordination of observables with the variants, even in the absence of scientific word knowledge of density. This relaxed scoring system meant that young children were not disqualified due to their developing scientific word knowledge; instead, the focus was on their reasoning and use of variables to think about causal phenomena, as previously used in Dündar-Coecke et al. (2019, 2020). The inter-judge reliability was further assessed *via* a colleague peer review, who scored six randomly chosen anonymized and age-blind scoresheets from each year group, resulting in a 100% agreement rate.

*2. A composite score for each causal domain* was computed to be used in the EQS model (shown in Figure 2). Children’s prediction (0–2), description (0–2), and explanation (0–3) scores were computed for sinking, absorption, and solution. Scores for each domain varied between 0 and 7.

## Results

### Does the Kind of Phenomena (Sinking, Absorption, and Solution) Present a Greater or Lesser Challenge to the Children?

#### Causal Tasks

**FIGURE 1:**
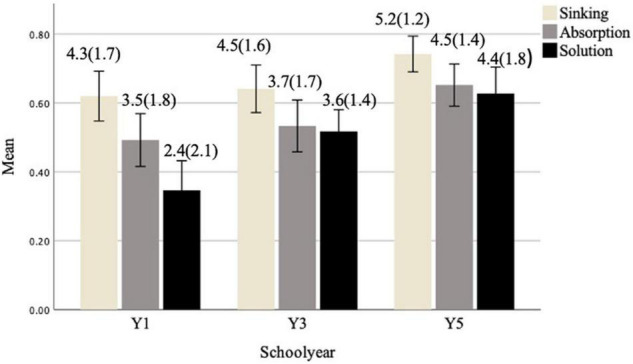
Response profile means for each causal measure by year groups, with standard deviations in parenthesis.

Figure 1 shows the response profiles for each age group on sinking, absorption and solution. All measures were normally distributed. Performance was consistently best on sinking across age groups, and worse for solution, indicating that sinking was easier for children from all age groups whilst solution was most difficult. However, absorption and solution means were similar in Y3 and Y5 groups.

A two-way mixed ANOVA (task within-subjects, age between-subjects) found a significant main effect of task, *F*(2, 90) = 15.74, *p* < 0.001, η*_*p*_*^2^ = 0.146. Bonferroni comparison showed a significant difference between scores on sinking, absorption and solution: sinking scores were significantly higher than absorption, and both were significantly higher than solution (*p* < 0.001 for all). Significant task by age interaction, *F*(2, 135) = 3.693, *p* = 0.045, η*_*p*_*^2^ = 0.035, showed that the growth in solution from Y1 toward Y3 was greater than the growth observed in sinking and absorption for the same age groups. There was also a main effect of age group in all tasks, *F*(2, 135) = 4.107, *p* < 0.05, η*_*p*_*^2^ = 0.057. For sinking, scores for Y1 were significantly lower than Y5, but no difference between Y1 and Y3. For absorption, Y1 scores were significantly lower than Y3 and Y5, with significant difference between Y3 and Y5. For solution, Y1 scores were significantly lower than Y3 and Y5; Y3 scores were also significantly lower than Y5.

One-way ANOVAs were used to test the differences between age groups on each prediction, description, and explanation score. Regarding sinking, there was a main effect of age on explanation, *F*(2, 135) = 4.367, *p* = 0.015, η*_*p*_*^2^ = 0.061, but only Y1 scores were significantly lower than Y5. There was no main effect of age on prediction or description, indicating that only Y1 and Y5 children differed in their explanations; the prediction and description profiles were similar across all age groups. Regarding absorption, there was a main effect of age on prediction *F*(2, 135) = 3.541, *p* = 0.032, η*_*p*_*^2^ = 0.050, and explanation, *F*(2, 135) = 7.143, *p* = 0.001, η*_*p*_*^2^ = 0.096, Y1 scores were significantly lower than both Y3 and Y5 for both, with no differences between Y3 and Y5. Regarding solution, there was a main effect of age on all scores: for prediction *F*(2, 135) = 5.205, *p* = 0.007, η*_*p*_*^2^ = 0.072, Y1 scores being significantly lower than Y5; for description, *F*(2, 135) = 6.784, *p* = 0.002, η*_*p*_*^2^ = 0.091, Y1 scores being significantly lower than Y3 and Y5 but no significant difference between Y3 and Y5; and for explanation, *F*(2, 135) = 10.838, *p* = 0.000, η*_*p*_*^2^ = 0.138, Y1 scores being significantly lower than Y3 and Y5, but no significant difference between Y3 and Y5.

Table 1 compares the highest and lowest scores of each component and shows that children’s prediction and description scores were consistently highest for sinking. However, explanation scores were generally lower. A significant number of children (32.6%) did not provide any explanation for solution and therefore were scored as zero.

**TABLE 1:** Percentages of children obtaining minimum and maximum scores on causal phenomena at each level.

	Sinking prediction	Absorption prediction	Solution prediction	Sinking description	Absorption description	Solution description	Sinking explanation	Absorption explanation	Solution explanation
**%**									
Minimum	18.8	39.9	26.8	8.7	13.1	26.1	8.7	16.7	32.6
Maximum	61.6	37.7	38.4	50.1	40.6	31.2	9.4	10.1	8.7

Looking at children’s total prediction, description, and explanation scores separately, 27.5% of children got scores of five or higher in prediction (max = 6 across three phenomena), 52.1% got scores of four or higher in description (max = 6 across three phenomena), but only 18.1% of them got scores of seven or higher in explanation (max = 9 across three phenomena), indicating that description responses were much better, but this did not guarantee a high level of explanation. Consistent with this, the majority of children mentioned weight in sinking (91.2%); softness in absorption (79.8%), and size in solution (86.1%), with some of them distinguishing between the differences in weight/softness/size as portrayed in the above table. The progress in description and explanation was largely attributable to differences between Y1 and Y5 children’s performances, as one-way ANOVAs found significant increases between those two groups, with Welch robust statistic for description = 5.075 (*df* = 2,89.446, *p* = 0.008); for explanation = 14.586 (*df* = 2, 89.110, *p* = 0.000). ANOVAs did not find a significant difference between the scores of Y3 and Y5 children. This implies that better explanation production is achieved sometime after Y1.

#### General Cognitive Measures

Both verbal tasks—WASI vocabulary and verbal analogies were normally distributed, whereas non-verbal Block design positively skewed due to Y1 and Y3 age groups having a longer tail, with skewness of 0.82 (*SE* = 0.20), indicating later growth in non-verbal competences (*p* < 0.05). One-way ANOVAs found significant increases with age on all tasks. For WASI vocabulary, Welch robust statistic was = 73.314 (*df* = 2, 88.992); for Verbal Analogies, it was 101.730 (*df* = 2, 88.048); for block design, it was 37.261 (*df* = 2, 85.254), with significant differences between all three age groups on both measures (*p* < 0.001 for all). Variance did not particularly decrease for any measure: for vocabulary, overall mean was 30.11 (*sd* = 6.76), for verbal analogies, it was 91.36 (*sd* = 12.77), and for block design, overall mean was 19.08 (*sd* = 10.73).

#### Correlations

The shape of the relations between the two verbal measures and the three phenomena was linear, but the scores on block design exhibited logarithmic relationship, explaining greater variance (with *R2* for linear fit = 0.839; *R2* for logarithmic fit = 0.935). Therefore, further analyses included the logarithmic transformation of block design.

Zero-order correlations demonstrated that general cognitive measures correlated with all variables (Table 2). Cognitive measures highly correlated to each other. The correlation between sinking and solution was weaker, and it became non-significant when age was controlled for. When age in months was controlled for, non-verbal (log) block design showed a stronger correlation with the three phenomena than vocabulary, except for solution. Solution consistently correlated better with the WASI vocabulary and Verbal Analogies, as opposed to non-verbal (log) block design. Schools did not correlate with any of the measures and were therefore excluded from the further analyses. Verbal Analogies and sinking highly correlated even when age was controlled for.

**TABLE 2:** Zero-order (above diagonal) and partial correlations (below diagonal) between causal measures and cognitive ability measures (significant associations in bold).

	Sinking	Absorption	Solution	Vocabulary	(Log)Block design	Verbal analogies
Sinking		0.491***	0.197*	0.350***	0.439***	0.439***
Absorption	0.459**		0.295***	0.292**	0.438**	0.342**
Solution	0.114	0.216*		0.453***	0.387***	0.468***
Vocabulary	0.267**	0.166	0.262**		0.637***	0.838***
(Log) Block design	0.382***	0.367***	0.200*	0.339***		0.809***
Verbal analogies	0.407***	0.234**	0.269**	0.674***	0.688***	

*p < 0.05, **p < 0.01, ***p < 0.001.

#### Regression Models

Hierarchical regression models examined the unique variance accounted for by verbal and non-verbal ability measures (see Table 3). Taking each causal component, age in months was entered in the first, WASI vocabulary in the second, Verbal Analogies in the third, and block design at the fourth stage to assess whether their effects were distinct from each other. *F* and Adjusted *R*^2^ change (Δ*R*^2^) values for each step was shown in Table 3.

**TABLE 3:** Hierarchical regression models of the relationships between cognitive ability and causal measures as dependent variables (*N* = 138).

	Step 1		Step 2		Step 3		Step 4	
	*F(df 1, 136)*	Δ*R*^2^	*F(df 2, 135)*	Δ*R*^2^	*F(df 3, 134)*	Δ*R*^2^	*F*(*4, 133)*	Δ*R*^2^
	*Age* (β)		Vocabulary (β)		Verbal analogies (β)		Log Block design (β)	
Sinking	7.97** 0.235**	0.055**	9.42*** 0.355**	0.067**	12.12*** 0.610***	0.090***	9.965*** 0.233	0.019
Absorption	9.548** 0.256**	0.066**	6.791** 0.220	0.026	5.923*** 0.327**	0.026*	8.005*** 0.476***	0.077***
Solution	27.029*** 0.407***	0.166***	19.361*** 0.328**	0.057**	13.799*** 0.234	0.013	10.327*** 0.053	0.001
Prediction	11.228*** 0.276***	0.076***	6.235** 0.125	0.008	10.859*** 0.679***	0.111***	11.522*** 0.426***	0.062***
Description	9.939** 0.248**	0.062**	6.796** 0.237*	0.030*	5.183** 0.228	0.013	4.864*** 0.264	0.024
Explanation	27.797*** 0.412***	0.170***	30.830*** 0.520***	0.144***	22.715*** 0.313*	0.024*	17.310*** 0.124	0.005

*p < 0.05, **p < 0.01, ***p < 0.001.

*Hierarchical regressions were run for each causal indices separately (i.e., sinking, absorption, etc.). The first value at the top shows the F values followed by the Beta values of the predictors underneath (i.e., age, vocabulary, verbal analogies, etc.). Adjusted R^2^ values for each association were shown under the ΔR^2^ column. So that readers can see the significant and non-significant models in a holistic fashion.*

This analysis produced significant models and final adjusted *R-squares*. For sinking, only verbal measures were significant predictors, while the non-verbal (log) block design was the strongest predictor for absorption. For solution, only WASI vocabulary measure was a significant predictor at the second stage, but the inclusion of block design consistently dropped the beta (β) values of all other variables. Age was consistently a significant predictor for all components.

Verbal Analogies explained the highest variance for prediction (β = 0.679; η*_*p*_*^2^ = 34.8); and another verbal measure WASI vocabulary explained the second highest variance for explanation (β = 0.520; η*_*p*_*^2^ = 41.6) scores. Otherwise, the predictive power of verbal and non-verbal cognitive measures altered across the domains.

The same analyses were conducted by using a composite measure involving both verbal ability tasks. The significances did not change, but the beta values of the composite verbal measure were slightly higher for each component.

### Which General Intelligence Mechanisms Are Associated With the Variance in Causal Reasoning, Specific to the Age Period Examined?

**FIGURE 2:**
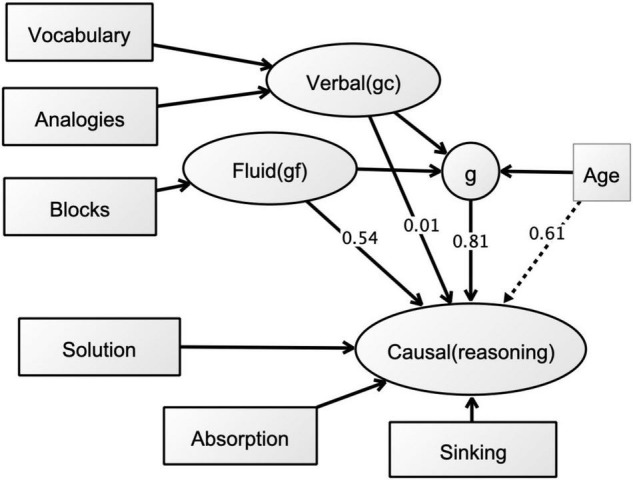
The EQS model investigating the patterns for causal explanatory thinking and general cognitive abilities (path coefficients are shown with numbers on each arrow; straight arrows show direct effects, dotted arrow shows the indirect effect).

The goodness-of-fits for complex relationships between causal and cognitive ability measures were tested using the EQS model as shown in Figure 2. The rationale for best-fitting function was based on the factor analytic model, where remaining free parameters were estimated using maximum likelihood solution. In the diagram, variables are represented by cycles, as they are residual factors, not observed measures [see e.g., Demetriou et al. (2017), for a similar paradigm using a structural equation model allowing testing of the possible differentiation of abilities with increasing *g* and age].

In the model, the three cognitive ability measures were regressed on the second-order *g* factor, as well as their residuals. This was to reveal the variance in each ability that is not accounted for by general ability (*g*) factor. This analysis showed that causal reasoning-*g* relations were high (0.814), and therefore *g* factor explained around 66% of the variance. Only non-verbal block design related significantly with causal reasoning, additionally to general ability (0.537) and explained a further 28% of the variance.

Goodness-of-fit indexes reflected a very good fit as shown in Table 4 (model Cronbach α = 0.769). Overall, the model suggested that the direct effect of g is 66% in causal reasoning. Of this, 37% was the indirect effect of age. The effect of gf captured by Block design was high, suggesting an additional 28%. The indirect effect of age suggested that relation between *g* and causal reasoning factors was age dependent, to a medium extent. In other words, more than half of the variance accounted for by the *g* factor was due to developmental differences in *g*.

**TABLE 4:** Fit indices for the EQS model.

	*N*	χ*^2^*	*df*	*AIC*	*CFI*	*RMSEA*
Model	138	13.470	10	−6.530	1.00	0.050

*χ^2^, chi-squared; df, degrees of freedom; AIC, Akaike’s information criterion; CFI, comparative fit index; RMSEA, root mean-square error of approximation.*

## Discussion

### Does the Kind of Phenomena (Sinking, Absorption, and Solution) Present a Greater or Lesser Challenge to the Children?

This question challenged analyses in three broad contexts; namely age effect, the involvement of domain-specific knowledge, and the variability in general cognitive abilities. Regarding the first, the data suggested a medium level of age effect on children’s causal explanation performances. This finding is partly in accordance with the literature, explained by both Piagetian and Vygotskian terms, where developmental mechanisms are driven by maturation and possibly *via* environmental interactions.

However, chronological and mental age may not always synchronize in mathematical terms. Several factors seem to play a role in the development of causal explanatory thinking. The data here showed that although the majority of children were able to predict and describe the phenomena relatively well, the qualitative aspects of their explanations differed significantly, particularly for sinking and solution. Overall, sinking was easier, while solution was most difficult for all age groups. Analyses of response levels for each phenomenon showed that the effect of a child’s age on his/her prediction or description performance in sinking was not significant. On the other hand, age had a significant effect on how children predicted and explained absorption, and at all levels of performance in solution. As discussed below, it is crucial to gain a full insight into this relatively early maturation in thinking about sinking, but later growth in solution, with absorption falling in the middle.

Distinct characteristics of the phenomena seem to matter, particularly the level of transparency in portraying visional and locational qualities in a more or less available manner to the observers, as also acknowledged in Rosen and Rozin’s (1993) study. Sinking portrayed the causal process in a more salient manner, because participants were able to observe the stone, grape, and water from the beginning to the end in an observable spatial-temporal layout. None of the objects disappeared, but their sinking rate evoked the involvement of intervening factors, such as gravity (and buoyancy for advanced thinkers). Unlike sinking, not all aspects of absorption were spatiotemporally laid out because although children were able to observe the rising water level, the mechanism behind upward motion required reasoning. For solution, spatial-temporal layout was least observable. The observed properties in the salt solution did not retain their quantities or qualities toward the end of the illustration, making it likely that either children’s anticipation of spatial-temporal layout became discordant with their common-sense, or it was harder for them to conceptualize the unobservable interactions as explicit forms. This finding supports the view discussed in the literature that causal explanatory systems are affected by certain kinds of foundational entities specific to each domain (Carey and Spelke, 1996; Sobel, 2004; Legare et al., 2009) and hence the nature of causal explanations can differ depending on domain characteristics (Wellman and Gelman, 1998; Keil et al., 2008). However, further qualitative investigations are needed to compare and clarify what children found difficult specific to each process.

Explanation competences evolved into higher maturity around the age of 7. The data showed that while the majority of Year 1 children relied largely on visual components when explaining causal phenomena, most Year 3 children were able to articulate abstract concepts and consider invisible factors at play. This ability became crucial for less transparent causal processes, like solution, which required children to shift from observable-dependent thinking to abstract conception. This would fall in line with the findings of Piaget and Inhelder’s (1974) study, looking at children’s understanding of the dissolution of sugar, which also suggested that children moved from pre-atomist to atomist thinking around a similar age.

It is not clear whether developmental changes in executive function and the emergence of rule-based thinking affected children’s causal explanation (as discussed Vosniadou and Brewer, 1987; Demetriou et al., 2010). Similarly, the effect of uneven conceptual development in different domains (Case, 1985; Demetriou et al., 1993, 2002; Carey, 2011), or slowly developing abilities playing a role in scientific reasoning (Dunbar and Klahr, 1988; Kuhn et al., 1988; Klahr, 2000; Klahr and Nigam, 2004; Dean and Kuhn, 2007; Kuhn, 2007; Zimmerman, 2007) warrant further exploration. Although the argument cannot be taken further on these points, the data here suggest that children’s ability to coordinate observed variables with unobserved variants in natural phenomena develops toward late childhood as an important component of the quality of their causal explanations. Notably, despite the large body of literature on executive function, to our knowledge no study to date has provided a developmental account exploring the link between executive function and causal reasoning. Further studies can investigate the relevance of this relationship, particularly the effect of maturation to executive function.

### Which General Intelligence Mechanisms Are Associated With the Variance in Causal Reasoning, Specific to the Age Period Examined?

As shown by hierarchical regression models, verbal measures were the only predictors for sinking and solution, while the non-verbal (block design) measure uniquely predicted absorption. However, these analyses suggested that none of the psychometric ability measures were independent, but instead the predictive power of verbal and non-verbal competences altered in each causal task. Given that verbal measures represented conceptual knowledge (know-what), while non-verbal measures corresponded to procedural knowledge (know-how), the high correlations between these measures can be explained using Karmiloff-Smith’s (1992) argument, proposing that words and their representations may be procedurally encoded, which highlights the link between conceptual and procedural understanding. In line with this argument, the alterations in the predictive power of these cognitive measures were weakly captured by the regressions, which suggested a mixed pattern, pointing to the possibility of multimodal parallel cognitive structures co-developing across the age range studied. This result is consistent with the proposal that cognitive systems are tuned to each other in multistructural and multisystemic ways, so that any change in one of the systems can affect the dynamics and help with co-processing amongst all of them across development (see Demetriou, 1993, for a review). Moreover, this result also complies with Spearman’s (1904) “positive manifold,” as discussed earlier.

On the other hand, the attempts to disentangle *g* from specific ability factors on causal understanding, as shown by the EQS model in Figure 2 indicated that, overall, there was a large psychometric ability factor in children’s causal explanations, with *g* impacting at a medium level, and *gf*, measured by block design, standing out as a unique factor beyond the *g* factor. Note that the psychometric *g* subsumed the variances of both verbal measures—vocabulary and verbal analogies. Despite this, the psychometric *g* is regarded as being a factor that is responsible for the positive correlations between all cognitive tests, and posited as a driving mechanism for reasoning, problem solving and here for causal explanatory thinking; *gf* as a non-verbal ability measure remained as a unique factor. Altogether, the psychometric *g* and *gf* explained 57% of the variance in causal thinking. Age—as an indirect effect- accounted for about 37% of the variance which highlighted the developmental differences affecting causal explanatory competences beyond general cognitive factors.

Spearman postulates that increasing g allows for increasing differentiation of specific cognitive systems, and therefore it might be the case here that higher mental power invests more variance in strengthening relations between specific cognitive abilities in causal reasoning. The data indeed showed how the contribution of verbal and non-verbal systems varied, and that their relation to the g gradually increased with age, and in turn causal inference qualitatively improved with age. Note that causal reasoning requires awareness of missing information and motivates a search for the unknown, indicating that there are phase-specific and ability-specific changes in this process, reflecting higher flexibility of the g (see Demetriou et al., 1993 for a similar outcome).

A counter argument could see age as a part of domain-specific mechanisms. However, the data show that this may not be so straightforward, as changes in the developmental processes tended to synchronize with changes in general ability, resulting in qualitative and quantitative changes in children’s causal explanations, enabling them to devise more abstract constructs in their causal thinking as they grow. There is also a possibility that abilities may become more/less closely related with childhood age as explained by the age differentiation-dedifferentiation hypothesis This hypothesis examines why abilities are differentially related across ages (age differentiation-dedifferentiation) and across levels of functioning (ability differentiation). Another hypothesis takes into account the diversity in neural sources mediating growth and considers age-related changes in the efficiency of neurotransmission as leading to age-related increases in ability dependence during childhood, while opposite patterns are observed during adulthood, where age-related decline in ability levels as associated with ability interrelations (see Tucker-Drob, 2009). The present study is limited to cross-sectional data, however, further studies could aim to overcome methodological shortcomings with samples containing participants from a wide range of age groups.

## Conclusion

Children’s causal explanations were associated with different cognitive ability factors dissimilarly across the year groups. Each task brought into focus unique conceptual and procedural representations that needed to be understood *via* intertwined routes, where psychometric abilities predicted causal reasoning substantially, but not entirely during the age periods studied. Demands from general ability factor increased or decreased depending on the task requirements. For instance, when the causal phenomenon included more abstract representations, as experienced in solution, mental operations were verbally more demanding. Confirmatory factor analyses showed that cognitive abilities matter to a large extent independently of age, with fluid reasoning standing out as a unique factor. However, this is the first study to explore the patterns between causal reasoning and types of cognitive ability. Further research can advance our understanding of whether there are other factors at play that can explain the nature of causal reasoning and uneven domain profiles.

## Data Availability Statement

The raw data supporting the conclusions of this article will be made available by the authors, without undue reservation.

## Ethics Statement

The studies involving human participants were reviewed and approved by the Institute of Education, UCL Research Committee (full ethical approval: REC 897). Written informed consent to participate in this study was provided by the participants’ legal guardian/next of kin.

## Author Contributions

SD-C conceptualized and developed the idea, conducted the research and analyses, wrote the manuscript.

## Conflict of Interest

SD-C was employed by Quantum Brain Art – QBA.

## Publisher’s Note

All claims expressed in this article are solely those of the authors and do not necessarily represent those of their affiliated organizations, or those of the publisher, the editors and the reviewers. Any product that may be evaluated in this article, or claim that may be made by its manufacturer, is not guaranteed or endorsed by the publisher.
