# Supplementary material for: To What Extent Is General Intelligence Relevant to Causal Reasoning? A Developmental Study
Source: Front Psychol. 2022 May 6;13:692552. doi: 10.3389/fpsyg.2022.692552 (PMC9159513; doi:10.3389/fpsyg.2022.692552)
Supplement: Supplementary file 1 [file Table_1.DOCX]

Supplementary Material

**1 Causal task scripts**

For *sinking*, children saw a stone and a grape of similar size but differing density, which sank at different rates in a half-meter tall transparent jar of still water. Children needed to predict outcomes ahead of witnessing simultaneous demonstrations of the two instances, then were asked to describe e.g., the rates of sinking, and to explain the outcomes. This functioned as a measure of causal inference assessing identification of basic factors (e.g. softness/hardness/heaviness of the materials), operative variables (e.g. relative weight of the materials) and mechanisms (e.g. object density, the role of water). A typical testing session involved the following scripts:

Experimenter: “I have got these two objects here: a stone and a grape. Do you want to hold them?” (A pause) “I am going to drop them in the water. What do you think will happen?”

Child: (predictions)

Experimenter: “Do you think that the same thing will happen to both objects?”

Child: (predictions)

Experimenter: “Thank you. Please watch carefully now. I will drop the objects and you will watch.” The experimenter drops the items together at the same time and asks: “Did you notice anything, what?”

Child: (descriptions)

Experimenter: “Did the same thing happen to both objects?”

Child: (descriptions)

Experimenter: “Thank you. Why do you think things happened that way?”

Child: (explanations)

Experimenter: “Do you think there might be another reason for that?”

For *absorption*, children saw water rising from a petri dish through strips of tissue and blotting paper of the same length/width, the water rising faster through the more open structure of the tissue. Similar to the sinking protocol, children needed to predict outcomes ahead of witnessing simultaneous demonstrations of the two instances, then were asked to describe e.g. the rates of absorption, and to explain the outcomes, as a measure of causal inference assessing identification of basic factors (e.g. softness/hardness of the materials), operative variables (e.g. relative softness/hardness/porousness of the materials) and mechanisms (e.g. relative size of the holes allowing water to rise up). A typical testing session involved the following scripts:

Experimenter: “I have got these two strips of paper here: a tissue and a blotting paper. Do you want to touch them?” (A pause) “I am going to dip them in the water. What do you think will happen?”

Child: (predictions)

Experimenter: “Do you think that the same thing will happen to both papers?”

Child: (predictions)

Experimenter: “Please watch carefully now. I will dip the papers and you will watch.” The experimenter dips the end of the papers in water together at the same time and asks: “Did you notice anything, what?”

Child: (descriptions)

Experimenter: “Did the same thing happen to both papers?”

Child: (descriptions)

Experimenter: “Why do you think things happened that way?”

Child: (explanations)

Experimenter: “Do you think there might be another reason for that?”

For *solution*, children saw the same small quantities of table and rock salt dissolve in warm water. The small quantity of the salt was assured with two equally very small spoons. The greater surface area to volume of the table salt led to more rapid solution. Similar to sinking and absorption protocols, children needed to predict outcomes ahead of witnessing simultaneous demonstrations of the two instances, then were asked to describe e.g. the rates of absorption, and to explain the outcomes, as a measure of causal inference assessing identification of basic factors (e.g. softness/hardness of the materials), operative variables (e.g. relative softness/hardness/compactness of the materials) and mechanisms (e.g. relative size of the grains allowing water to penetrate in). A typical testing session involved the following scripts:

Experimenter: “I have got these two kinds of salts here: table salt and rock salt. I am going to drop a tiny piece from each in the water. What do you think will happen?”

Child: (predictions)

Experimenter: “Do you think that the same thing will happen to both kinds of salt?”

Child: (predictions)

Experimenter: “Thank you. Please watch carefully now. I will drop the salts and you will watch.” The experimenter drops the samples of the salts together at the same time and asks: “Did you notice anything, what?”

Child: (descriptions)

Experimenter: “Did the same thing happen to both kinds of salt?”

Child: (descriptions)

Experimenter: “Why do you think things happened that way?”

Experimenter: “Do you think there might be another reason for that?”

**2 Causal tasks scoring system**

The three-stage scoring structure. The following tables were used to check with the inter-judge reliability of the scoring system.

Table 1

Scoring system for causal tasks

| Component | Sinking | Absorption | Solution |
| --- | --- | --- | --- |
| Prediction from prior knowledge  (0-2) | 1=Correct prediction for stone (i.e., sinks)  1=Correct prediction for difference between stone and grape (e.g., sink at different speeds) | 1=Correct prediction for tissue paper  1=Correct prediction for difference between tissue and blotting  paper | 1=Correct prediction for table salt  1=Correct prediction for difference between table and rock salt |
| Description of observation  (0-2) | 1=Correct description for stone  1=Correct description for grape | 1=Correct description for tissue paper  1=Correct description for blotting paper | 1=Correct description for table salt  1=Correct description for rock salt |
| Explanation/  inference  (0-3) | 0=No explanation  1=Weight or size without difference between objects  2=Weight or size with difference  3=Use of any factors and variables (density, heavy/light of its size) applied to causal mechanism | 0=No explanation  1=Thickness/softness/texture without difference between types of paper  2=Thickness/softness/texture with difference  3=Use of any factors and variables (more/less space, holes affecting absorbency) applied to mechanism | 0=No explanation  1=Grain/size without difference between types of salt  2=Grain/size with difference  3=Use of any factors and variables (grain/size with surface area) applied to mechanism |

Table 2

Examples for the level of explanations

| Phenomena | Level 1 | Level 2 | Level 3 |
| --- | --- | --- | --- |
| Sinking | “They are heavy, and they sank to the bottom” | ‘The stone is heavier than the grape, so they sank to the bottom differently” | “They are both heavier than the water and cannot hold air in it, so they sank to the bottom. But the stone sank quicker than the grape because it’s got more stuff in it so the water can’t hold it up as it did to grape.” |
| Absorption | “If you dip the paper in the water, they get wet because they’re soft” | “The tissue paper is thinner than the other paper, so water rises faster in it” | “The tissue paper has holes in it that help water to rise up. Water holds on the walls of the holes and layers and that helps it to climb up. Other paper has some space in it, but not as much as the tissue paper.” |
| Solution | “They go into water because they’re small and spread out” | “The table salt is smaller than the rock salt, so it disappears quicker.” | “The size of the two types of salt is different. And this is rockier so water cannot go into it easily. They both dissolve in the water, but rocky one takes more time than the table salt.” |
